# Inhibiting function of human fetal dermal mesenchymal stem cells on bioactivities of keloid fibroblasts

**DOI:** 10.1186/s13287-017-0624-0

**Published:** 2017-07-18

**Authors:** Ya Jiao, Xiao Wang, Jixun Zhang, Yongjun Qi, Hongmin Gong, Duyin Jiang

**Affiliations:** 1 Department of Emergency and Department of Burns and Plastic Surgery, The Second Hospital of Shandong University, Jinan, Shandong 250033 People’s Republic of China; 2 School of Medicine, Shandong University, Jinan, Shandong 250012 People’s Republic of China

**Keywords:** Fetal dermal mesenchymal stem cells, Keloid, Conditioned medium

## Abstract

**Background:**

Keloid is one kind of benign skin disease caused by hyperplasia of fibroblasts and collagen fibrils. It is refractory due to the lack of an effective treatment at present, which puts pressure on seeking a new therapeutic regimen. Mesenchymal stem cells (MSCs) from fetal skin are considered to play a crucial role in scarless healing. Nevertheless, the efficacy of them in keloid disorders remains poorly understood.

**Methods:**

Keloid fibroblasts (KFs), human adult dermal fibroblasts (ADFs), and human fetal dermal mesenchymal stem cells (FDMSCs) were isolated to single cells and cultured in Dulbecco’s modified Eagle’s medium (DMEM). ADFs and FDMSCs were used to generate ADF-conditioned medium (A-CM) and FDMSC-conditioned medium (F-CM). The effects of A-CM and F-CM on KFs were tested using MTT assay, BrdU assay, TUNEL assay, quantitative polymerase chain reaction, Western blot, and annexin V-FITC/PI binding assay,.

**Results:**

FDMSCs inhibited the bioactivity of KFs, downregulated the expression of the antiapoptotic protein BCL-2, and upregulated the expression of the proapoptotic protein BAX of KFs by secreting some soluble substances, thus accelerating the apoptosis of KFs.

**Conclusion:**

F-CM induces apoptosis of KFs, providing a novel treatment strategy for keloid disorders.

**Electronic supplementary material:**

The online version of this article (doi:10.1186/s13287-017-0624-0) contains supplementary material, which is available to authorized users.

## Background

As one kind of benign skin disease, keloid is caused by hyperplasia of fibroblasts and collagen fibrils [1]. Keloids can grow excessively and invade nearby healthy skin, so the patient with keloid suffers from great physical and psychological pressure [2]. Although keloid is a clinically frequently occurring disease, it is refractory due to the lack of an effective treatment. Therefore, there is a pressing need to seek a new therapeutic regimen. Recent research has indicated that mesenchymal stem cells (MSCs) play significant roles in scarless wound healing and tissue regeneration [3–5]. In addition, fetal skin cells are thought to play key roles in fetal scarless healing [6, 7]. MSCs from fetal skin are considered to play a crucial role in this process. Nevertheless, the efficacy of them in keloid remains poorly understood.

In the present study, we investigated the effects of fetal dermal MSC (FDMSC)-conditioned medium (F-CM) on keloid fibroblasts (KFs). Our studies revealed that FDMSCs could induce the apoptosis of KFs by secreting some soluble substance and we further verify the feasibility of new treatments.

## Results

### F-CM induces the apoptosis of KFs

**Fig. 1:**
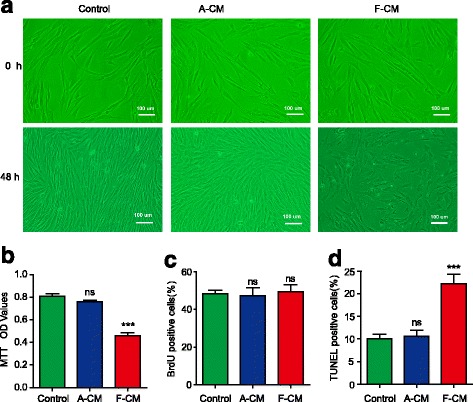
The impact of F-CM and A-CM on KF morphology (**a**), living cell number (**b**), proliferation (**c**), and apoptosis (**d**). The fetal dermal MSC-conditioned medium (*F-CM*) group had less living cells and more apoptotic cells than the control group or adult dermal fibroblast-conditioned medium (*A-CM*) group. ***P < 0.001, vs. the control group. *ns* not significant, *OD* optical density

As we know, morphology is the most direct representation of the cell state. Cells with good growth status have excellent transparency, high refraction, and a clear boundary. On the contrary, cells in bad condition have inferior transparency, low refraction, and an unclear boundary, and even lose the characteristics of the original cells. As a control group, KFs cultured with serum-free medium (SFM) showed spindle or triangular shapes with a clear boundary, which had excellent transparency and high refraction. KFs cultured with adult dermal fibroblast (ADF)-conditioned medium (A-CM) looked similar to the control group with high cell density and an orderly arrangement (Fig. 1a). As for the F-CM group, we observed a decreased cell density, increased number of floating cells, and the characteristic apoptotic appearance including cell shrinkage, chromatin condensation, membrane blebbing, formation of apoptotic bodies, and disordered cell arrangement [8] (Fig. 1a). Meanwhile, the number of living KFs in the F-CM group was much less than in the control group, which was not embodied in the A-CM group (Fig. 1b). Furthermore, we detected the effects of F-CM and A-CM on the proliferation and apoptosis of KFs; neither A-CM nor F-CM had any significant effect on KF proliferation (Fig. 1c). However, the percentage of TUNEL-positive cells in the F-CM group was 22.24 ± 2.05%, much higher than in the control group and A-CM group (Fig. 1d), indicating that F-CM promoted the apoptosis of KFs. Taken together, these data suggest that F-CM inhibits the bioactivity of KFs by mainly inducing cell apoptosis.

### F-CM effects the expression of BCL-2 and BAX

It is well known that the antiapoptotic protein BCL-2 inhibits cell apoptosis by blocking cytochrome C release from mitochondria. In various kinds of tumor cells, the expression of BCL-2 is upregulated [9, 10]. The proapoptotic protein BAX could form heterodimers with BCL-2, and suppress the antiapoptotic effect of BCL-2. As a result, the upregulation of BCL-2 promotes apoptosis [11]. The ratio of BCL-2 expression to BAX expression is a direct index of cell apoptosis. To investigate the molecular mechanism of F-CM on KFs, quantitative real-time polymerase chain reaction (qPCR) and Western blot were performed.

**Fig. 2:**
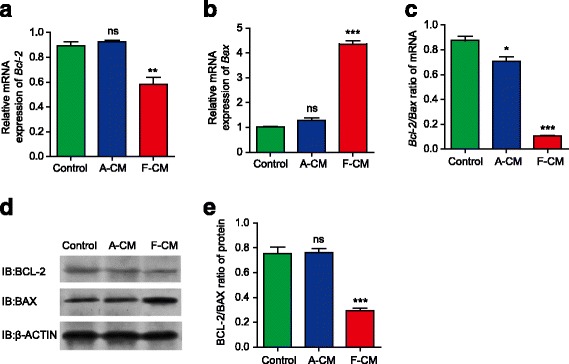
The expression of apoptosis-associated genes and proteins analyzed with qPCR (**a–c**) and Western blot (**d**,**e**). has the effect of promoting the expression of proapoptotic genes and proteins and inhibiting expression of antiapoptotic genes and proteins. *P < 0.05, **P < 0.01, ***P < 0.001, vs. control group. *A-CM* adult dermal fibroblast-conditioned medium, *ns* not significant

As shown in Fig. 2a, the mRNA level of *Bcl-2* was downregulated in the F-CM group while the A-CM group showed no significant difference compared with the control group. Meanwhile, the RNA level of Bax was upregulated in the F-CM group while the A-CM group showed only a very slight change compared with the control group (Fig. 2b). As a result, the *Bcl-2/Bax* ratio of the F-CM group was much lower than the control group (Fig. 2c), which indicates that the F-CM group has more apoptotic cells. Interestingly, the *Bcl-2/Bax* ratio of the A-CM group was a little lower than that of the control group. We suspected that ADFs absorbed some nutrients and released some metabolic waste into the A-CM, which also happened in the experimental study of others [12]; the cell medium must be replaced regularly to avoid affecting the survival status of cells. Consistently, the protein level of BCL-2 was downregulated, and the protein level of BAX was upregulated in the F-CM group. However, there was no significant change in BCL-2 and BAX protein levels in the A-CM group (Fig. 2d), which was inconsistent with the mRNA levels. Translation of individual mRNA species into their encoded proteins is regulated producing discrepancies between mRNA and protein levels, which may resulted from altered translational efficiencies [13, 14]. Thus, the BCL-2/BAX ratio of the F-CM group was notably downregulated (Fig. 2e). To summarize, F-CM downregulates BCL-2 expression and upregulates BAX expression of KFs, resulting in KF apoptosis.

### F-CM accelerates the late phase of KF apoptosis

To further investigate the impact of F-CM on KFs, an Annexin V-fluorescein isothiocyanate (FITC)/propidium iodide (PI) binding assay was performed. In living cells the cell membrane is impermeable to V-FITC and PI. In early apoptotic cells, phosphotidylserine is translocated to the extracellular surface of the cell membrane. Annexin V-FITC specifically binds with phosphotidylserine. However, the cell membrane of early apoptotic cells is still impermeable to PI. In late apoptotic cells, the cell membrane is ruptured and permeable to V-FITC and PI. In dead cells the cell membrane is destroyed completely and stained with PI only [15]. We could distinguish and quantitatively determine the percentage of dead cells (Annexin V-FITC-negative/PI-positive), viable cells (Annexin V-FITC-negative/PI-negative), early apoptotic cells (Annexin V-FITC-positive/PI-negative), and late apoptotic cells (Annexin V-FITC-positive/PI-positive).

**Fig. 3:**
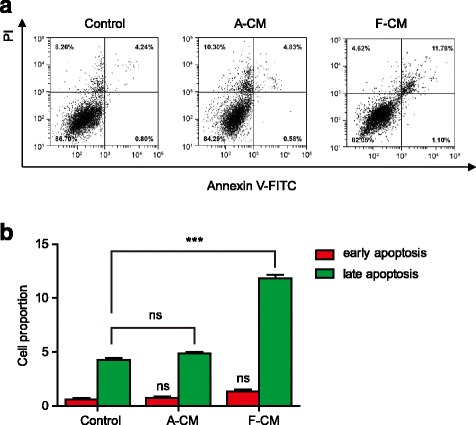
Flow cytometry analysis of KFs. **a** Scatter plots of fluorescein isothiocyanate (*FITC*)-fluorescence versus propidium iodide (*PI*)-fluorescence in the three groups. **b** The proportion of early and late apoptosis cells in the three groups, which implied that F-CM could mainly induce late apoptosis of KFs. ***P < 0.001, vs. control group. *A-CM* adult dermal fibroblast-conditioned medium, *ns* not significant

Our research revealed that there were more apoptotic cells in the F-CM group than the A-CM group and the control group (Fig. 3a). Moreover, the proportion of late apoptotic cells was significantly increased as shown in Fig. 3b, indicating that F-CM mainly induced the late phase of apoptosis.

## Discussion

The etiology of keloid is unknown, and the complexity of its development without specific factors being identified has caused difficulties in finding effective treatment. The main histopathologic features of keloid are extracellular matrix (ECM) degradation and collagen remodeling. These processes are regulated by the matrix metalloproteinases (MMPs) with significantly elevated activity and increased expression in KFs [16]. MicroRNA-205-5p of KFs, which is known to induce cell apoptosis, inhibit cell invasion and migration, and impair cell viability, is downregulated in KFs [17]. In addition, many reports have demonstrated that KFs, as one of the primary research targets, were the key cellular mediators of fibrogenesis. The role of KFs in keloid is attracting particular attention for a therapeutic effect.

In addition to some traditional therapies, there are many new therapeutic approaches, such as stem cell therapy, that have brought new hopes to managing keloid. Recent studies show that MSCs can regulate the wound-healing process and prevent scar formation in keloid formation, suggesting their promising therapeutic role in keloid. The paracrine role of MSCs is also attracting particular attention for their therapeutic effect on wound healing, tissue repair, and scar remodeling [18–20]. Reports have demonstrated that the use of adipose-derived MSCs could facilitate cutaneous wound healing and form a thinner scar, and the underlying mechanisms involved are the downregulated expression of transforming growth factor (TGF)-β1, decreased accumulation of collagen, and increased expression of decorin [21]. Bone marrow-derived MSCs pathologically suppressed excessive skin fibrosis, inhibited KF proliferation, and ECM deposition through TGF-β3-dependent activation, and adult tissue-derived MSCs are candidates for cellular therapy to promote scarless wound healing by attenuating inflammation [22]. Furthermore, MSC transplantation therapy has shown safety in effectively promoting skin wound healing in recent years.

In contrast to the adult, fetal wound healing can happen and repair skin wounds with the absence of scar formation in the early and middle embryo stage of mammals, which provides a potential therapeutic implication for human adult wounds. Although, the exact mechanisms of scarless fetal wound healing remain largely unknown, they are thought to be mainly due to the special properties of the embryo [23, 24]. Fetal wound healing is characterized by the attenuated inflammation with fewer proinflammatory cytokines, and the fetal ECM being rich in type III collagen and hyaluronan plays an important role in scarless wound healing as well [25]. Moreover, other differences between fetal skin cells and postnatal skin cells, such as gene expression, cellular mediators, and stem cell function, contribute to the different outcomes [26, 27].

Based on the research results above, we subsequently selected the intersection of MSCs and fetal skin cells as our breakthrough point: FDMSCs. We hypothesized that FDMSCs could regulate the bioactivity of KFs. To verify our hypothesis, we designed experiments to demonstrate the function of FDMSCs. KFs were incubated with A-CM, F-CM, and SFM. We found that F-CM significantly induces cell apoptosis of KFs. Consistent with the effect of F-CM, coculture of FMSCs and KFs also induced the apoptosis of KFs (Additional file 1: Figure S1). Stem cell transplantation is not at our disposal now owing to undiscovered characteristics of stem cells. Application of the conditioned medium is a better choice.

FDMSCs show superiority, including low immunogenicity, easy expansion in vitro, and higher proliferation potential and differentiation capability compared with stem cells from other sources. FDMSCs were not recognized by T lymphocytes after transplantation and immunological rejection was avoided. Therefore, FDMSCs show significant promise for their clinical application for safety and effectiveness [28]. In order to further verify the safety of F-CM, we have performed BrdU double staining with MSC markers to detect whether renewing KFs changed their nature. The results showed that BRDU-positive cells were negative for MSCs markers (Additional file 1: Figure S9). The renewing KFs were not transdifferentiated under F-CM conditions, which provides further evidence of its safety. In addition, time-dependent effects of F-CM were observed which showed that the ratio of cell death rose in a time-dependent manner; just like with clinical drugs, clinical treatment needs appropriate concentration, administration time, and duration.

## Conclusion

Our research revealed that FDMSCs downregulated the expression of the antiapoptotic protein BCL-2 and upregulated the expression of the proapoptotic protein BAX of KFs by secreting soluble substances. Therefore, FDMSCs accelerates the apoptosis of KFs, providing novel treatment strategies for keloid.

## Methods

### Tissue procurement

All tissues were obtained from patients of The Second Hospital of Shandong University. Fetal skin tissues were taken from 16–20 week human fetuses which were accidental abortions without hereditary disease or drug therapy. Adult skin tissues were obtained from patients who underwent cosmetic surgery (surgical waste). Keloid tissues were taken from keloid patients confirmed by pathology without any treatment in the previous 3 years. There were five specimens for each sample at least, and all samples were stored at 4 °C in phosphate-buffered saline (PBS; Invitrogen, USA) containing 100 U/ml penicillin-streptomycin (Gibco, USA).

### Cell isolation and cell culture

Fetal skin tissues from the dorsum were carefully dissected into 1.0-cm^2^ pieces and digested with 0.25% dispase (Sigma, USA) overnight at 4 °C to remove the epidermis completely. Tissues were then cut into 0.1-cm^2^ pieces and digested with 0.125% type I collagenase (Worthington, USA) for 5 min at 37 °C to obtain FDMSCs. The adult skin tissues were cut into 1.0-cm^2^ pieces and digested with 0.25% dispase overnight at 4 °C. Tissues were then cut into 0.1-cm^2^ pieces and digested with 0.125% type I collagenase for 30 min at 37 °C to obtain ADFs. The isolated cells were cultured in low-glucose Dulbecco’s modified Eagle’s medium (DMEM; HyClone, USA) containing10% fetal bovine serum (FBS; Gibco, USA) and 1% 100 U/ml penicillin-streptomycin. Keloid tissues without epidermis were minced into pieces as small as possible and then placed in the culture dishes. Two hours later, the minced keloid tissues were cultured in low-glucose DMEM with 10% FBS and 1% 100 U/ml penicillin-streptomycin.

FDMSCs and ADFs attached to the bottom of the dish 12 h later. After 1 or 2 weeks, KFs migrated from the tissue pieces. All of the cells were cultured at 37 °C with 5% CO_2_. When cells reached 70–80% confluence, they were digested with 0.25% trypsin (Sigma, USA) and subcultured. Only 4–6 generations of cells were used for experiments. These cells had no obvious difference in appearance and exhibited a spindle or triangular shape like fibroblasts. When cells reached 90% confluence they exhibited an interesting morphology, like vorticity or fish school (Additional file 1: Figure S2).

### Detection of surface antigens

The slides of cells were cultured in six-well plates (Corning, USA), fixed, permeabilized, and then immunostained with primary antibodies overnight at 4 °C. Cell slides were then incubated with species-specific fluorochrome-conjugated secondary antibodies (Cell Signaling Technology, USA) for 1 h at room temperature and shielded from light. Finally, the images were obtained by fluorescence microscopy coupled with a camera (Nikon Eclipse TS100, Japan) and NIS-Elements software (Nikon, Japan). The MSC phenotypes of FDMSCs were characterized by flow cytometry analysis.

The antibodies anti-CD44, anti-CD90, anti-CD105, anti-CD14, anti-CD34, anti-CD45, anti-SSEA-4, anti-OCT-4, anti-vimentin, and anti-CK19 were purchased from Cell Signaling Technology, USA.

FDMSCs were positive for MSC markers including CD44, CD90, and CD105 (Additional file 1: Figure S3 and Figure S5) but negative for hematopoietic stem cell markers including CD14, CD34, and CD45 (Additional file 1: Figure S4 and Figure S5). Interestingly, FDMSCs expressed embryonic markers including SSEA-4 and OCT-4 (Additional file 1: Figure S6). All cells were positive for the dermal cell marker vimentin (Additional file 1: Figure S7) and negative for the epidermal cell marker CK19 (Additional file 1: Figure S8).

### Multilineage differentiation potential of FDMSCs

FDMSCs were plated into six-well plates and cultured with osteogenic differentiation medium (Cyagen, China) when cells reached 70% confluence. The culture medium was changed every 3 days. After 5–7 days, calcium nodules appeared. After 20 days of differentiation, the cells were stained with alizarin red (Cyagen, China). As shown in Additional file 1 (Figure S10), the ECM was rich in calcium, confirming osteogenic differentiation.

FDMSCs were plated into six-well plate and cultured in medium. When cells reached 100% confluence, the medium was changed to adipogenic differentiation medium (Cyagen, China). After 3 days, the medium was replaced with adipogenic maintenance medium (Cyagen, China) and, 24 h later, the medium was replaced with adipogenic differentiation medium again. Five to seven days later, the intracellular lipid vacuoles appeared. After four cycles, the cells were stained with Oil-red O (Cyagen, China). As shown in Additional file 1 (Figure S10), more than 70% of the cells were positive for Oil-red O, confirming adipogenic differentiation.

FDMSCs were plated into six-well plate and cultured in medium. When cells reached 70% confluence, the medium was changed to chondrogenic differentiation medium (Cyagen, China). Under the induction of chondrogenic differentiation medium, the morphology of cells were changed gradually from the vorticity pattern to a chaotic pattern and lost polarity. After about 20 days, the cellular layer shrank and differentiated into adipocytes which could be stained with Alcian blue (Cyagen, China), confirming their chondrogenic phenotype as shown in Additional file 1 (Figure S10).

### Preparation of conditioned medium

When FDMSCs and ADFs reached 80% confluence in 12-well plates (Corning, USA), cells were cultured in serum-free medium (SFM) overnight. Then cells were cultured with 1 ml SFM per well. F-CM and A-CM were harvested after 48 h incubation. The conditioned medium was then filter-sterilized through a 0.22-μm Millex-GP syringe filter (Millipore, USA) and stored at –80 °C.

### Morphology of KFs

KFs were seeded into a six-well plate at 2 × 10^4^ cells/well density and grown in SFM for 24 h. SFM was replaced by A-CM or F-CM as compared with the control group (SFM group), and the cells were cultured for 48 h before being observed by inverted microscope.

### MTT assay

Cells were seeded in 96-well plates (Corning, USA) at a density of 3 × 10^3^ cells/well and cultured in SFM for 24 h, and then incubated with F-CM, A-CM, or SFM separately for 48 h at 37 °C in a 5% CO_2_ humidified atmosphere. 3-(4,5-dimethyl-2-thiazolyl)-2,5-diphenyl-2-H-tetrazolium bromide (MTT; Amresco, USA) solution was added to each well to a final concentration of 0.5 mg/ml. MTT is reduced to purple formazan in living cells which can be solubilized with dimethyl sulfoxide (DMSO; Solarbio, China). The optical density (OD) for each well was measured at reference wavelength of 492 nm using a microplate reader (Thermo Fisher Scientific, USA) to evaluate cell viability.

### BrdU assay

Cells were plated in six-well plates at a concentration of 2 × 10^3^ cells/well. After incubation with F-CM, A-CM, or SFM for 48 h, 5-bromo-2-deoxyuridine (BrdU; SCBT, USA) was added to the medium to a final concentration of 10 μmol/ml. Two hours later, KFs were stained with BrdU and DAPI, according to the directions of the BrdU immunofluorescence kit (SCBT, USA). For each group, five random fields were counted to calculate the percentage of BrdU and DAPI double-positive cells.

In order to verify if renewing KFs were induced into MSCs by F-CM, BrdU double staining with MSCs markers including CD44, CD90, and CD109 was performed.

### TUNEL assay

KFs were incubated with TUNEL (Beyotime, China) for 1 h at 37 °C followed by DAPI for the identification of nuclei. For each group, five random fields were counted to calculate the percentage of TUNEL and DAPI double-positive cells.

### Transwell coculture assay

In order to observe the direct effects of FDMSCs on KFs, transwell coculture of FDMSCs with KFs was performed. FDMSCs were seeded into the upper chamber while KFs were seeded into the lower chamber. After FDMSCs and KFs were separately cultured in transwell coculture system with 0.4-μm pore size inserts (Corning, USA) over 48 h, KFs were prepared for the following experiments including MTT assay, BrdU cell proliferation assay, and TUNEL apoptosis assay.

### Quantitative real-time polymerase chain reaction

Total RNAs were extracted from KFs using Trizol Reagent (Invitrogen, CA, USA) and reverse-transcribed using a SuperScript III First-Strand Synthesis Super Mix Kit (Invitrogen). The primers used in the qPCR to detect the mRNA levels are as follows: 1) forward primer 5’-TGGATGACTGAGTACCTGAACCG-3’ and reverse primer 5’-TGAGCAGAGTCTTCAGAGACAGC-3’ for human *B-cell lymphoma-2* (*Bcl-2*; BGI, China). 2) forward primer 5’-ACTGGACAGTAACATGGAGCTG-3’ and reverse primer 5’-AGCCCATGATGGTTCTGATCAG-3’ for human *Bcl2-associated X gene* (*Bax*; BGI, China). and 3) forward primer 5’- GGCACCCAGCACAATGAAG -3’ and reverse primer 5’-GCCGATCCACACGGAGTACT-3’ for human *β-Actin* (BGI, China).

### Western blot

Cells were lysed in RIPA lysis buffer (Beyotime, China) at 4 °C for 30 min. Protein extracts were applied to 10% SDS-polyacrylamide gel electrophoresis (SDS-PAGE) and transferred to a nitrocellulose membrane (Bio-Rad, USA). The membranes were incubated with primary antibodies against BCL-2 (1:1000 dilution; Cell Signaling Technology, USA), BAX (1:1000 dilution; Cell Signaling Technology), or β-actin (1:1000 dilution; Cell Signaling Technology) overnight at 4 °C and then with secondary antibodies coupled to horseradish peroxidase (HRP; 1:3000 dilution; Cell Signaling Technology) for 1 h at room temperature. Band signals were detected with an enhanced chemiluminescence (ECL) system (Thermo Fisher Scientific, USA) and then visualized.

### Annexin V-FITC/PI binding assay

According to the instructions of the annexin V-FITC/PI flow cytometric assay kit (Invitrogen, USA), KFs were harvested, washed, and suspended at a density of 1.0 × 10^6^ cells/ml with binding buffer containing FITC-conjugated annexin V and PI. The cells were then mixed and incubated at room temperature for 15 min. We obtained and analyzed the scatter plots of FITC fluorescence versus PI fluorescence by flow cytometry.

### Statistical analysis

All of the data were analyzed by Student *t* test (unpaired) by Statistical Product and Service Solutions (SPSS, IBM Corp., USA). The data are shown as the mean ± standard deviation (SD) from at least three independent repeated experiments.

## Additional file

Additional file 1: Figure S1. Direct effect of ADFs and FDMSCs on KFs. ***:*P*<0.001, ns: no significance. **Figure S2.** Morphology and characterization of KFs, ADFs, and FDMSCs. **Figure S3.** Immunofluorescent staining showed that FDMSCs were positive for the mesenchymal stem cell markers CD44, CD90, and CD105. **Figure S4.** Immunofluorescent staining showed that FDMSCs were negative for the hematopoietic stem cell markers CD14, CD34, and CD45. **Figure S5.** Flow cytometry showed that FDMSCs were positive for the mesenchymal stem cell markers CD44, CD90, and CD105 (A, B, C) and negative for the hematopoietic stem cell markers CD14, CD34, and CD45 (D, E, F). **Figure S6.** FDMSCs expressed embryonic markers including SSEA-4 and OCT-4. **Figure S7.** Immunofluorescent staining showed that KFs, ADFs, and FDMSCs were positive for the dermal cell marker vimentin. **Figure S8.** Immunofluorescent staining showed that KFs, ADFs, and FDMSCs were negative for the epidermal cell marker CK19. **Figure S9.** Immunofluorescent staining and BrdU staining showed that F-CM did not alter the nature of KFs. **Figure S10.** Multilineage differentiation potential of FDMSCs. Alizarin red staining for osteocytes, Oil-red O staining for adipocytes, and Alcian blue staining for Chondrocytes. (DOCX 1210 kb)

## Abbreviations

A-CM: Adult dermal fibroblast-conditioned medium
ADF: Adult dermal fibroblast
Bax: Bcl2-associated X
Bcl-2: B-cell lymphoma-2
BrdU: 5-Bromo-2-deoxyuridine
DMEM: Dulbecco’s modified Eagle’s medium
DMSO: Dimethyl sulfoxide
ECM: Extracellular matrix
FBS: Fetal bovine serum
F-CM: Fetal dermal mesenchymal stem cell-conditioned medium
FDMSC: Fetal dermal mesenchymal stem cell
FITC: Fluorescein isothiocyanate
KF: Keloid fibroblast
MSC: Mesenchymal stem cell
MTT: 3-(4,5-dimethyl-2-thiazolyl)-2,5-diphenyl-2-H-tetrazolium bromide
OD: Optical density
PI: Propidium iodide
qPCR: Quantitative real-time polymerase chain reaction
SD: Standard deviation
SFM: Serum-free medium
TGF: Transforming growth factor
