# Supplementary material for: Inhibiting function of human fetal dermal mesenchymal stem cells on bioactivities of keloid fibroblasts
Source: Stem Cell Res Ther. 2017 Jul 18;8:170. doi: 10.1186/s13287-017-0624-0 (PMC5516368; doi:10.1186/s13287-017-0624-0)
Supplement: Additional file 1: Figure S1. — Direct effect of ADFs and FDMSCs on KFs. ***:P<0.001, ns: no significance. Figure S2. Morphology and characterization of KFs, ADFs, and FDMSCs. Figure S3. Immunofluorescent staining showed that FDMSCs were positive for the mesenchymal stem cell markers CD44, CD90, and CD105. Figure S4. Immunofluorescent staining showed that FDMSCs were negative for the hematopoietic stem cell markers CD14, CD34, and CD45. Figure S5. Flow cytometry showed that FDMSCs were positive for the mesenchymal stem cell markers CD44, CD90, and CD105 (A, B, C) and negative for the hematopoietic stem cell markers CD14, CD34, and CD45 (D, E, F). Figure S6. FDMSCs expressed embryonic markers including SSEA-4 and OCT-4. Figure S7. Immunofluorescent staining showed that KFs, ADFs, and FDMSCs were positive for the dermal cell marker vimentin. Figure S8. Immunofluorescent staining showed that KFs, ADFs, and FDMSCs were negative for the epidermal cell marker CK19. Figure S9. Immunofluorescent staining and BrdU staining showed that F-CM did not alter the nature of KFs. Figure S10. Multilineage differentiation potential of FDMSCs. Alizarin red staining for osteocytes, Oil-red O staining for adipocytes, and Alcian blue staining for Chondrocytes. (DOCX 1210 kb) [file 13287_2017_624_MOESM1_ESM.docx]

Additional file 1:

**Inhibiting function of Human Fetal Dermal Mesenchymal Stem Cells on Bioactivities of Keloid Fibroblasts**

**Figure S1.**

**
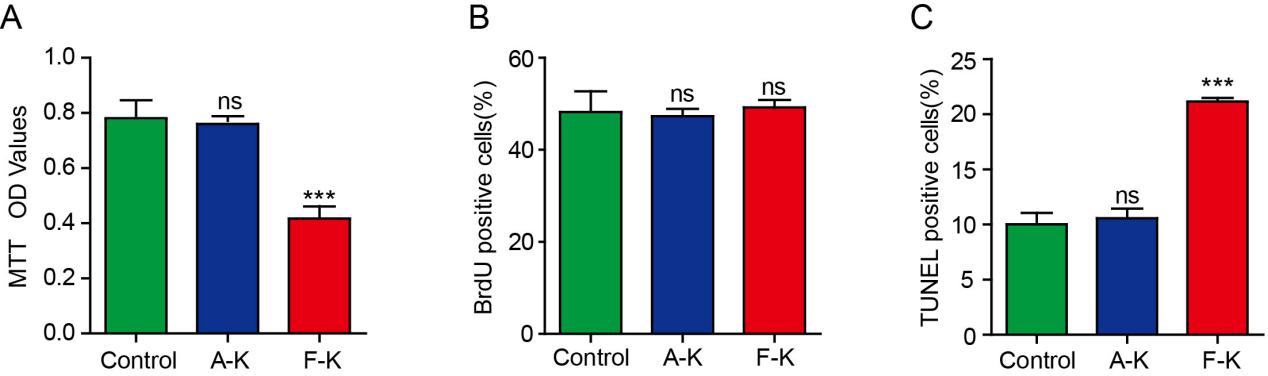
**

**Figure S1. Direct effect of ADFs and FDMSCs on KFs. “ns” indicated no significance vs. control group. “***”** **indicated statistically significant difference at P<0.001 vs. control group.** In transwell co-culture system with 0.4μm pore size inserts, ADFs or FDMSCs were plated in the upper compartment, meanwhile, KFs were plated in the lower compartment. (A) By MTT assay, FDMSCs decreased the number of living KFs, however, ADFs had no effect on it. (B) By BrdU assay, FDMSCs and ADFs had no effect on KFs proliferation. (C) By TUNEL assay, FDMSCs induced the apoptosis of KFs, and ADFs had no effect on it.

**Figure S2.**

**
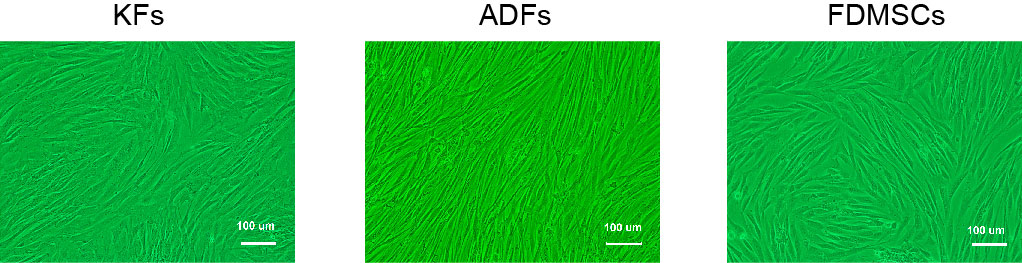
**

**FigureS2. Morphology and characterization of KFs, ADFs and FDMSCs.** Three kinds of primary cells like marked on photographs, exhibited spindle or triangular shape like fibroblasts.

**Figure S3.**

**
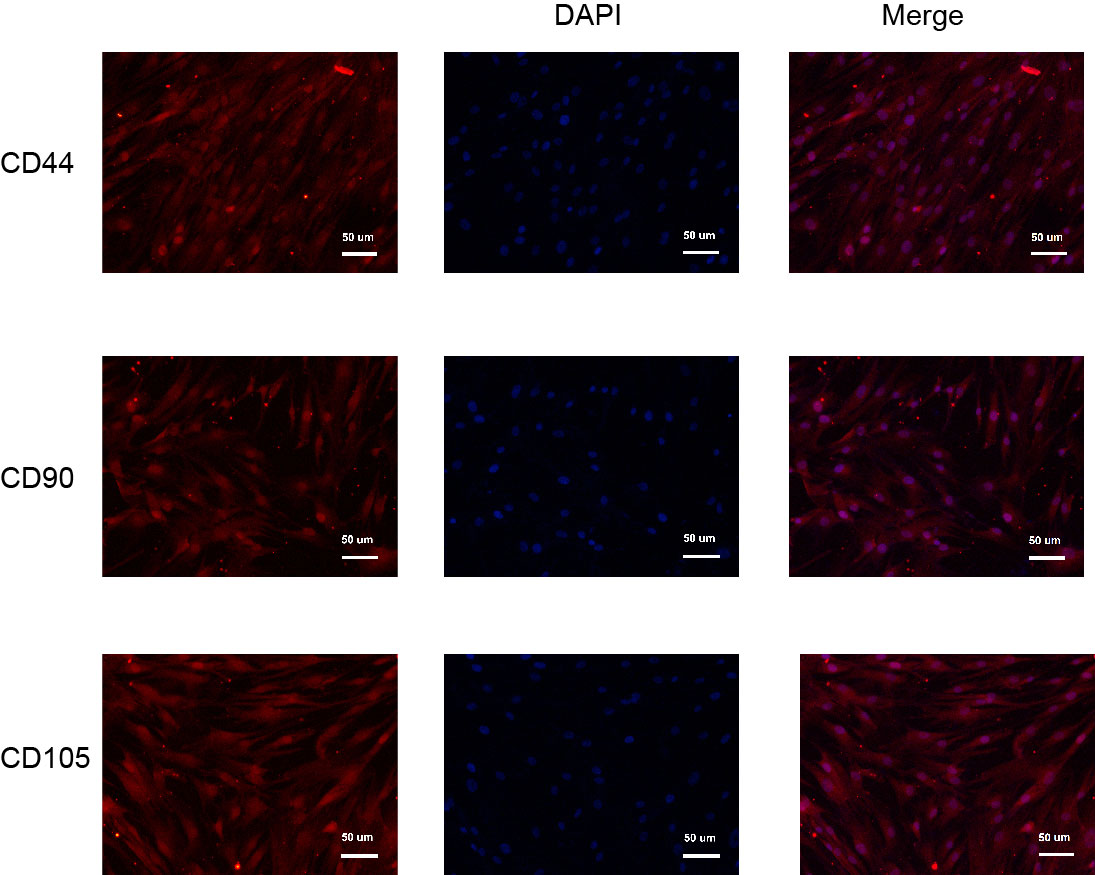
**

**Figure S3. Immunofluorescent staining showed that FDMSCs were positive for mesenchymal stem cell markers CD44, CD90 and CD105** ^[1]^**.**

**Figure S4.**

**
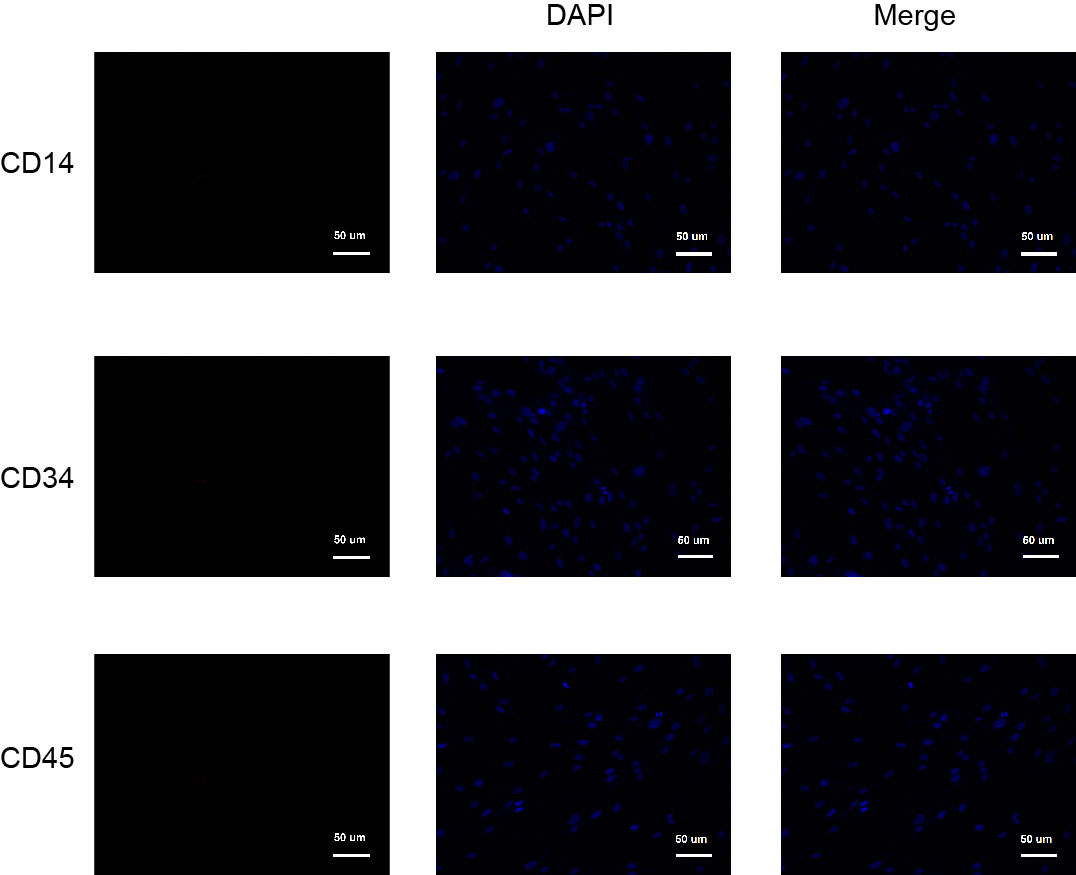
**

**Figure S4. Immunofluorescent staining showed that FDMSCs were negative for hematopoietic stem cell markers CD14, CD34 and CD45** ^[1]^**.**

**Figure S5.**

A B


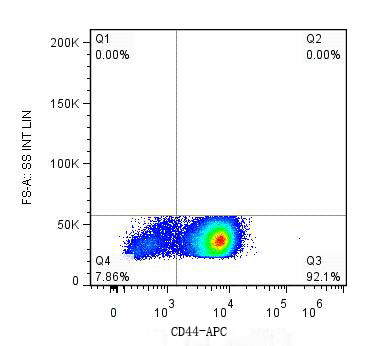

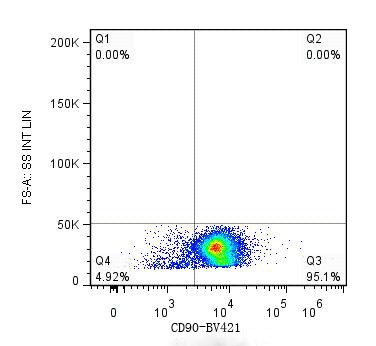


C D

**
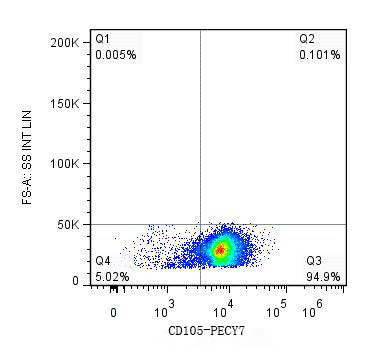

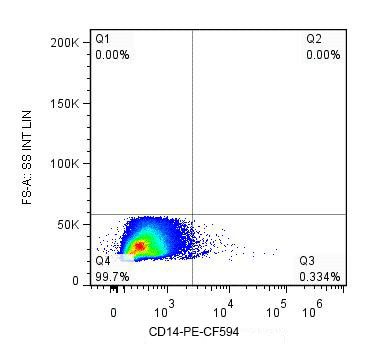
**

E F

**
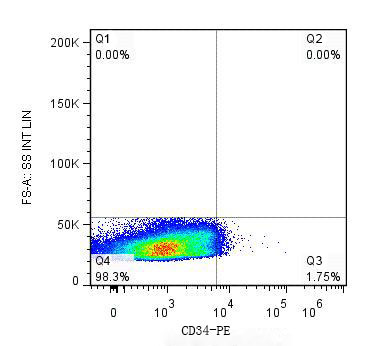

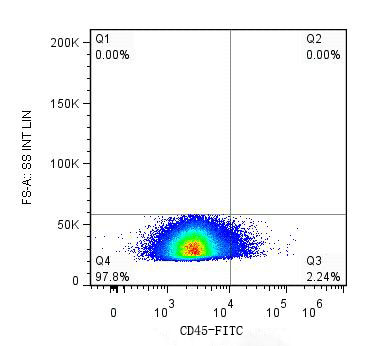
**

**Figure S5. Flow cytometry showed that FDMSCs were positive for mesenchymal stem cell markers CD44, CD90, CD105 (A, B, C) and negative for hematopoietic stem cell markers CD14, CD34, CD45 (D, E, F)** ^[1]^**.**

**Figure S6.**

**
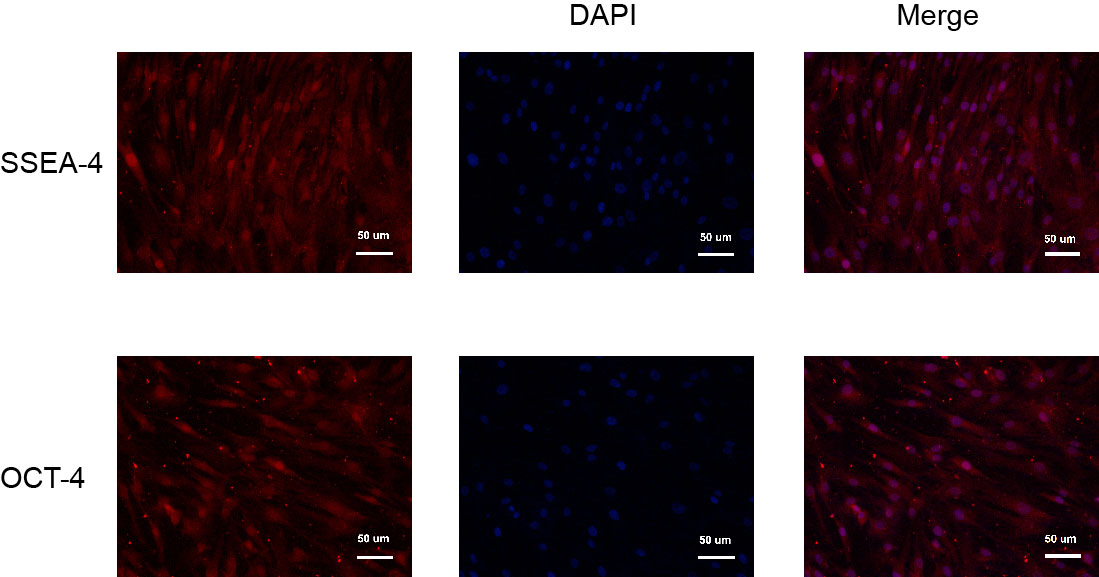
**

**Figure S6. FDMSCs expressed embryonic markers including** **SSEA-4 and OCT-4.**

**Figure S7.**

**
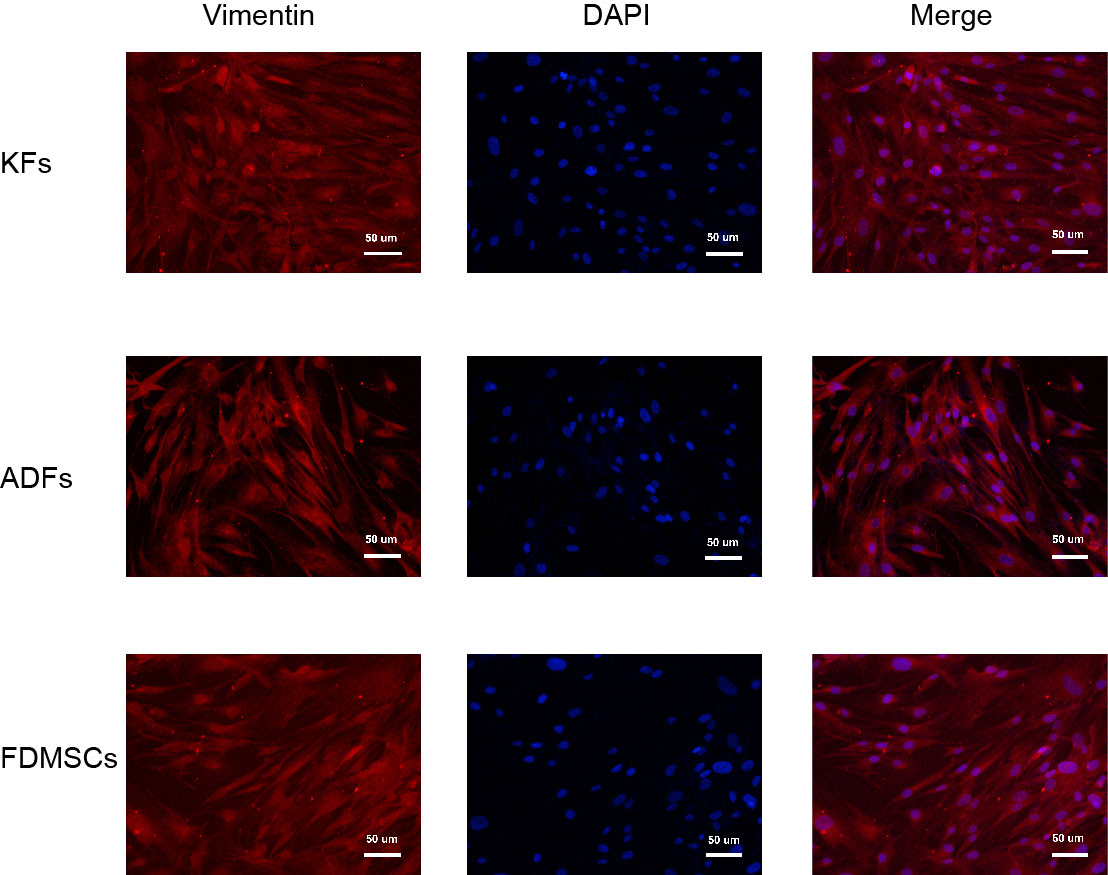
**

**Figure S7. Immunofluorescent staining showed that KFs, ADFs and FDMSCs were positive for dermal cell marker vimentin.**

**Figure S8.**

**
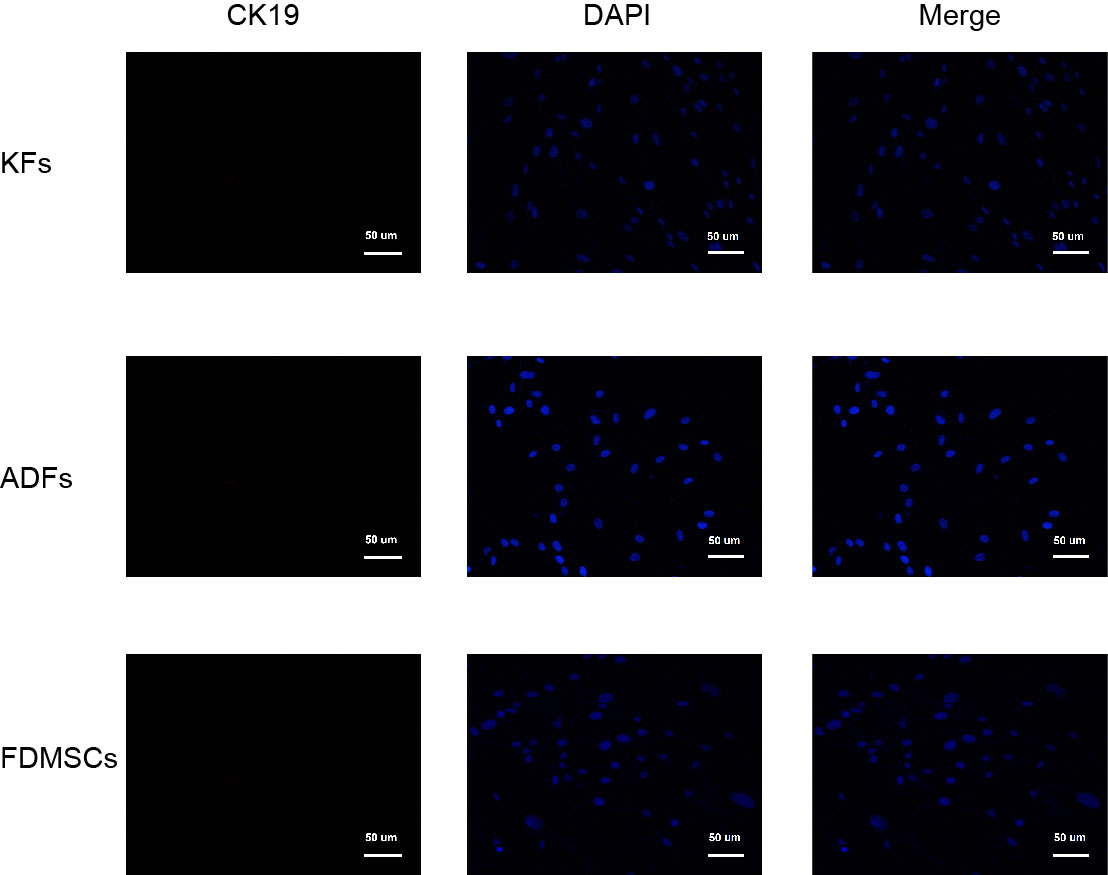
**

**Figure S8. Immunofluorescent staining showed that KFs, ADFs and FDMSCs were negative for epidermal cell marker CK19.**

**Figure S9.**

**
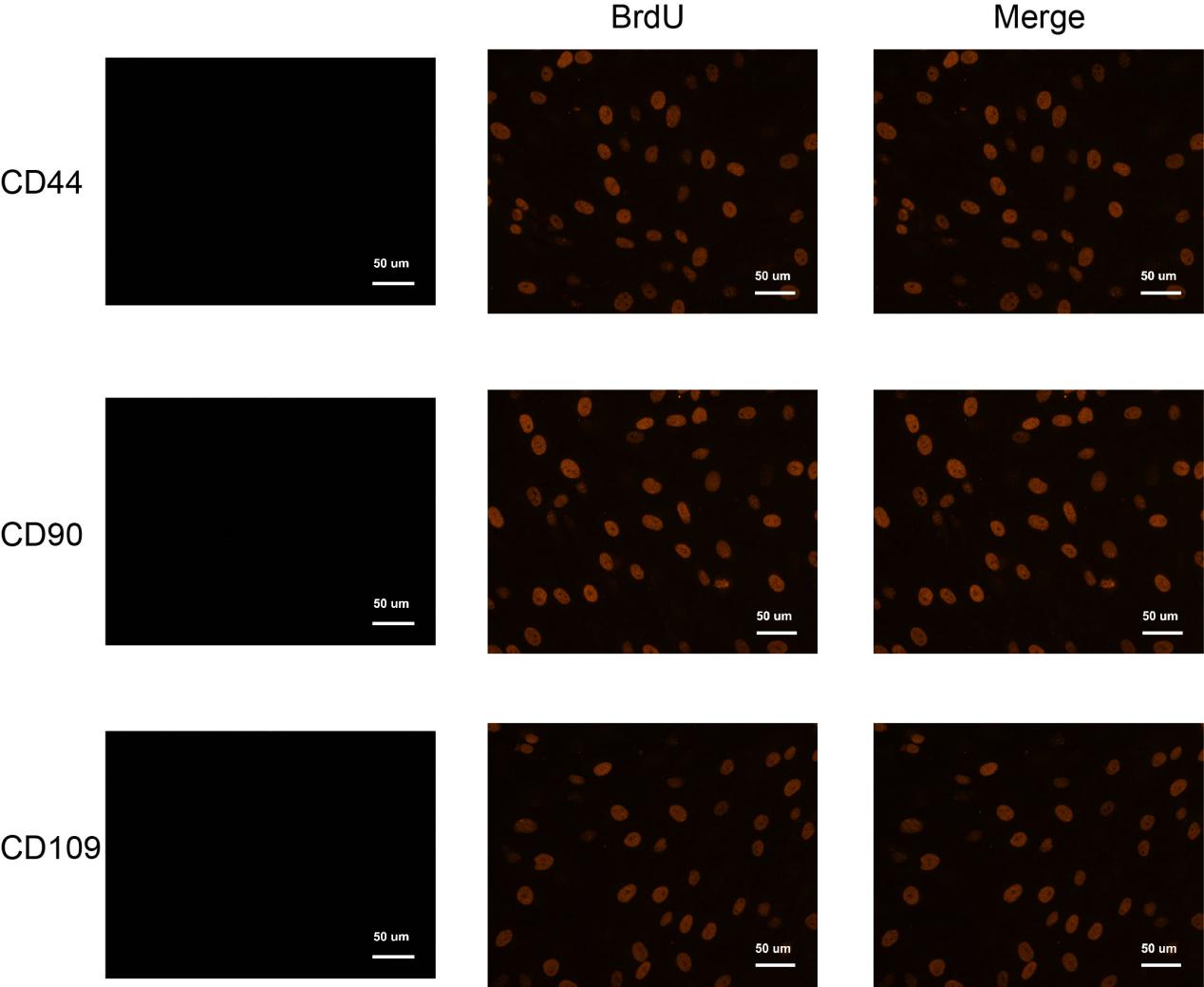
**

**Figure S9. Immunofluorescent staining and BrdU staining showed that F-CM did not alter the nature of KFs.**

**Figure S10.**

**
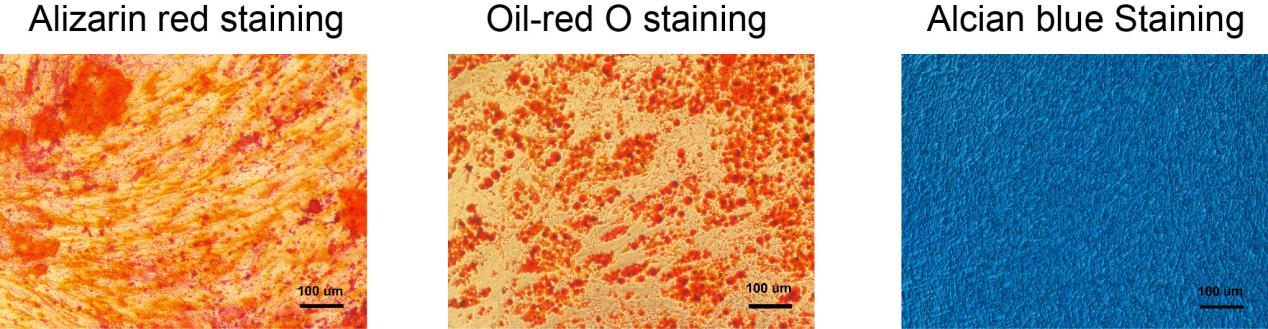
**

**Figure S10. Multilineage differentiation potential of FDMSCs.** Alizarin red staining for osteocytes, Oil-red O staining for adipocytes, and Alcian blue staining for Chondrocytes.

**References**

[1] Bocelli-Tyndall C, Bracci L, Spagnoli G, et al. Bone marrow mesenchymal stromal cells (BM-MSCs) from healthy donors and auto-immune disease patients reduce the proliferation of autologous- and allogeneic-stimulated lymphocytes in vitro[J]. Rheumatology (Oxford). 2007, 46(3): 403-408.
